# Vitamin B12 is Low in Milk of Early Postpartum Women in Urban Tanzania, and was not Significantly Increased by High dose Supplementation

**DOI:** 10.3390/nu12040963

**Published:** 2020-03-31

**Authors:** Omar N. Lweno, Christopher R. Sudfeld, Ellen Hertzmark, Karim P. Manji, Said Aboud, Ramadhani A. Noor, Honorati Masanja, Nahya Salim, Setareh Shahab-Ferdows, Lindsay H. Allen, Wafaie W. Fawzi

**Affiliations:** 1 Ifakara Health Institute, Bagamoyo Research and Training Center, P.O. Box 74 Bagamoyo, Tanzania; hmasanja@ihi.or.tz; 2 Department of Global Health and Population, Harvard T.H. Chan School of Public Health, Boston, MA 02115, USA; csudfeld@hsph.harvard.edu (C.R.S.); stleh@channing.harvard.edu (E.H.); ramanoor@gmail.com (R.A.N.); mina@hsph.harvard.edu (W.W.F.); 3 Muhimbili University of Health and Allied Sciences, P.O. Box 65001 Dar-es-Salaam, Tanzania; kpmanji@gmail.com (K.P.M.); aboudsaid@yahoo.com (S.A.); nsalim@gmail.com (N.S.); 4 USDA ARS Western Human Nutrition Research Centre, University of California Davis, CA 95616, USA; setti.shahab-ferdows@usda.gov; 5 Program in International and Community Nutrition, Department of Nutrition, University of California, Davis, CA 95616, USA; Lindsay.allen@ars.usda.gov

**Keywords:** vitamin B12, postpartum, breast milk, supplementation, Urban Tanzania

## Abstract

The effect of maternal multivitamin supplementation on breast milk vitamin B12 concentrations has not been examined in Tanzania, where the prevalence of maternal plasma B12 insufficiency is 25.6%. Multivitamins (containing 50 µg vitamin B12) or placebo were provided during pregnancy and in the postpartum period. Breast milk samples were collected at or around six weeks postpartum from 491 participants in a trial of multivitamins (NCT00197548). Linear and logistic regression models were used to examine the effect of supplements on vitamin B12 concentration in milk and its associations with other variables including potential confounders. Median vitamin B12 concentration in breast milk was 206 pmol/L and 70% of women had levels indicating inadequacy (<310 pmol/L). Multivitamin supplements did not significantly reduce the odds of inadequate vitamin B12 in breast milk, suggesting suboptimal absorption. A single unit increase in maternal hemoglobin at six weeks was associated with 18% lower odds of inadequate vitamin B12 in breast milk. Participants with higher BMI at baseline had double the odds of having inadequate vitamin B12 than the reference group (<22 kg/m^2^). Trials to determine the optimal dose, route, and duration of supplementation to improve maternal B12 status in Sub-Saharan Africa are of utmost importance.

## 1. Introduction

Vitamin B12 deficiency is common in low- and middle-income settings, with reported prevalence ranging from 40% in South America to 60%–80% in some parts of Asia and Africa [1]. In Tanzania, the prevalence of maternal vitamin B12 insufficiency measured in plasma was reported to be 25.6% [2]. Vitamin B12 deficiency may affect all age groups and is more common in areas where the intake of animal source foods is low [1]. Pregnant and lactating women have been noted to be at particularly high risk for vitamin B12 deficiency due to increased vitamin B12 and nutritional demands of the mother, the fetus and the infant during this period [3]. Adequate vitamin B12 in infants is important for their growth and cognitive development, and its deficiency leads to failure to thrive, developmental regression, and severe neuropathy [4]. The concentration of vitamin B12 in breast milk is correlated with maternal plasma vitamin B12 and maternal intake [5,6]. Longitudinal studies have demonstrated the progressive decline of breast milk vitamin B12 concentration during the first 3 months of lactation and by the 4th month of life the lower concentration of vitamin B12 in breast milk is associated with an increase in infant serum methylmalonic acid, which has been suggested as indicating that status is insufficient to maintain metabolic functions [7]. Several studies have demonstrated that women receiving vitamin B12 supplements have higher concentrations of plasma vitamin B12 during pregnancy [6,8], and the main predictor of vitamin B12 concentration in breast milk is maternal B12 status and stores during pregnancy [1]. Results from recent studies conducted in Kenya have shown that vitamin B12 concentrations in breast milk were not associated with reported dietary intake of vitamin B12, animal source food intake and household hunger [9]. Maternal supplementation with vitamin B12 has been reported to increase vitamin B12 in breast milk in India using a dose of 50 µg [6] and Canada where a supplement containing 12 µg was used [4]. In this secondary analysis of data, we examine the effect of vitamin B12 supplementation on vitamin B12 concentration in the milk of mothers in Dar es Salaam, Tanzania, at approximately six weeks postpartum. We also secondarily identify observational risk factors for low breast milk vitamin B12. We hypothesized that the use of micronutrient supplements containing vitamin B12 and, intake of animal source foods would reduce the risk of low vitamin B12 concentrations in milk.

## 2. Materials and Methods

The perinatal study (PNS) was a randomized, double-blind, placebo-controlled trial designed to investigate the effect of a multivitamin supplement on perinatal outcomes among human immunodeficiency virus (HIV) negative women in Dar-es-Salaam, Tanzania [10]. Details have been published elsewhere [10]. In brief, the inclusion criteria for the primary study were (1) HIV-negative pregnant women with a gestational age ranging from 12–27 weeks according to the date of the last menstrual period who attended study antenatal clinics between August 2001 and July 2004, (2) written informed consent to participate in the study, (3) a plan to stay in Dar es Salaam until delivery and for 1 year thereafter. The study participants were randomly assigned to receive a daily oral dose of either a multivitamin supplement or placebo from the time of enrolment until six weeks postpartum. The randomization list was prepared according to a randomization sequence in blocks of 20. The daily multivitamins contained vitamin B1 (20 mg), vitamin B2 (20 mg), vitamin B6 (25 mg), niacin (100 mg), vitamin B12 (50 µg), vitamin C (500 mg), vitamin E (30 mg), and folic acid (0.8 mg). The control arm received a daily placebo supplement. Blinding was achieved by using active and placebo tablets that were similar in shape, size, and color and were packaged in identical bottles.

All participants received antenatal care according to the Tanzanian guidelines that include daily supplemental iron (60 mg of elemental iron) and folic acid (0.25 mg) as well as malaria prophylaxis (Fansidar, Roche) at 20 weeks and 30 weeks which was the standard of care. In addition, women were given de-worming medication (Mebendazole), and they were treated for any diagnosed illnesses that happened during the antenatal follow-up.

The population in the breast milk vitamin B12 study included a total of 500 women who were randomly selected from a sub-sample of 3000 trial women who had milk collected at six weeks postpartum. A computer-generated list of random numbers was used to select samples for the secondary study. For budgetary reasons, we could only analyze 500 samples. With this number of samples and assuming a prevalence of 0.75 (as in Cambodia study), the minimum detectable difference in proportion of women having B12 inadequacy between multivitamin and placebo groups with power 0.69 would be 10%.

### 2.1. Data Collection

Socio-demographic data (education, marital status, occupation and wealth) were recorded during the screening visit including information on the recruitment site, date of birth, date of last menstrual period (LMP) for calculation of gestational age in completed weeks at entry, and pregnancy history including history of live births, stillbirths or abortion. At baseline, weight in kilograms, height in centimeters and mid-upper arm circumference in centimeters were also recorded. The wealth score was calculated using the Filmer-Pritchett method [11]. Maternal dietary data were collected using an 119 item adult food frequency questionnaire (FFQ) specifically designed for Tanzania. The adult food frequency questionnaire was used during pregnancy and repeated within 40 days post-partum. Among other things, the FFQ asked how often in the previous month the following animal-source food items were eaten by the mother: beef, goat, pork, liver, chicken, fried fish, fresh fish, sardines, dried fish and eggs. We also asked about the intake of other foods like margarine on bread, cow’s milk, and tea with milk, and vegetables. The last FFQ at or before the date of the B12 sample was used to assess the dietary intake of vitamin B12, animal protein, total protein and total energy intake before the measurement of milk vitamin B12 concentration at six weeks post-partum. Macronutrient (total protein, animal protein) densities were computed as percentage of energy from the macronutrient. Vitamin B12 intake was energy-adjusted [12]. The minimum dietary diversity for women (MDD-W) score was calculated using 24-h recalls and later on categorized into those with score less than 5 and those with 5 or more groups [13]. The MDD-W is a population-level assessment indicator of the proportion of women (15–49 years) who consume a diet containing 5 or more food groups, which can be used as a proxy indicator of higher micronutrient adequacy [13]. A score of less than 5 food groups is indicative of micronutrient inadequacy. Complete blood counts were analyzed with a CBC5 counter (Coulter Corp, Miami, FL) by technicians who were blinded to treatment allocation and were obtained at six weeks postpartum.

### 2.2. Breast Milk Sample Collection and Vitamin B12 Analysis

Twenty (20) mL of breast milk were collected in 50 mL plastic cryovials at six weeks postpartum. Women were instructed to breastfeed for 2 min before expressing breast milk in plastic cryovials (20 mL). The contents were mixed up by inverting the collecting tubes twice or thrice before being aliquoted and stored in containers at minus 4 degrees centigrade and protected from direct light. Women were allowed to breastfeed after sample collection. Samples were stored at −80 degrees centigrade until they were shipped for analysis. The shipment of samples from Muhimbili Teaching Hospital to the USDA Agricultural Research Service Western Human Nutrition Center in Davis, California, was done on dry ice following the accepted international procedures.

The analysis of milk samples was performed as described by Hampel and colleagues [14]. Breast milk samples were analyzed for vitamin B12 by the VB for IMMULITE 1000 solid-phase automated competitive binding chemiluminescent enzyme immunoassay (Siemens). The CV for the assay was 9%, which was calculated by running control samples with each set of samples. The concentration used to set the adequate intake (AI) is 310 pmol/L [9].

### 2.3. Statistical Analysis

Descriptive statistics using means and standard deviations were used for age, gestational age at randomization and at delivery, and anthropometric characteristics. Medians and interquartile range were used for skewed variables including dietary vitamin B12 and vitamin B12 concentration in breast milk. The Wilcoxon rank-sum (Mann-Whitney) test was used to compare median intake of vitamin B12, animal and total protein and calorie intake between the two trial arms. Proportions of categorical variables such as education, marital status, parity, wealth index score, and pregnancy history were compared using chi-square tests.

The distribution of breast milk vitamin B12 concentration was determined to be non-normal and was, therefore, log transformed for the analysis. Linear regression was used to identify predictors for the logarithmic vitamin B12 concentration in milk. Coefficients were exponentiated, multiplied by 100, then 100 was subtracted to compute the percent difference in B12 concentration given a unit increase in each predictor. Confidence intervals (CI) were computed in a similar manner. Logistic regression models were used to predict vitamin B12 concentrations below the AI value of 310 pmol/L. Covariates with *p* < 0.20 in univariate models were included in a multivariate model. Odds ratios (OR) and their 95% confidence intervals were computed. Study arm was automatically included in the multivariate linear and logistic models. *p*-values < 0.05 were considered statistically significant.

The dietary intakes of vitamin B12, animal and total protein were included in the multivariate model though they were not statistically significant in univariate analyses because we were interested to determine their relationships with milk vitamin B12 concentration. All analyses were done using STATA version 15 (Stata Corporation, College Station, TX, USA).

### 2.4. Ethics

Ethical approval to perform secondary analysis of data was granted by the National Research Ethics Committee (NathREC) in Tanzania (NIMR/HQ/R.8a/Vol.IX/2649) and the Harvard T.H.Chan School of Public Health Human Subjects Committee in Boston, MA. The IRB approval code for Harvard was 10433. The material transfer agreement (MTA) between the Muhimbili University of Health and Allied Sciences (MUHAS) and the Harvard T.H. Chan School of Public Health (HSPH) was signed by the Chair of the Medical Research coordinating committee before the shipment of breast milk samples.

## 3. Results

### 3.1. Study Participants

**Figure 1:**
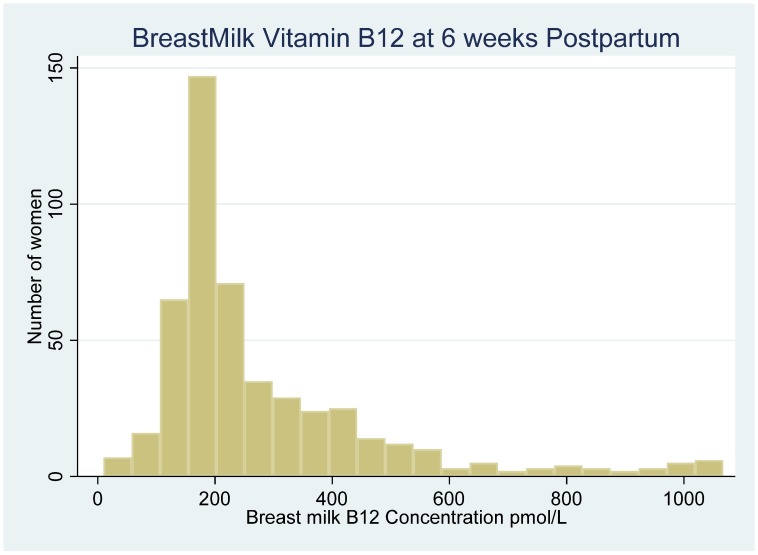
Distribution of Breast milk vitamin B12 concentrations at 6^th^ weeks postpartum visit among Human Immunodeficiency Virus (HIV) negative women in Urban Tanzania.

Participants in the primary study (PNS) were Tanzanians, mean age of 25 years who completed primary education (three-quarters) of participants, and the majority were pregnant for the first time (primigravida). Breast milk was collected from 500 mothers during their 6th week (mean = 5.4 weeks, SD = 0.5) post-partum visit between March 2003 and February 2005. Three samples were too small for analysis, four samples were taken more than 8 weeks after delivery, and two could not be linked to randomized study participants leaving 491 samples. The distribution of breast milk vitamin B12 concentration at six weeks postpartum visit is shown in Figure 1.

The two study arms were similar on measures of age, gestational age at randomization, adherence to study medication, and baseline anthropometric measurements (Table 1).

The groups were also similar in terms of a wealth score calculated using the Filmer-Pritchett method [11].

The mean minimum dietary diversity for women (MDD-W) score during pregnancy was 2.86 (SD = 2.66). Only 6 (1.3%) of the women assessed had a mean score of MDD-W of at least 5 food groups, which is the FAO definition of minimum dietary diversity [13]. The median vitamin B12 intake was above the recommended dietary allowance (RDA) of 2.8 µg/d for lactating women in the multivitamin and placebo groups [15].

### 3.2. Supplementation and Vitamin B12 Concentration in Breast Milk

**Figure 2:**
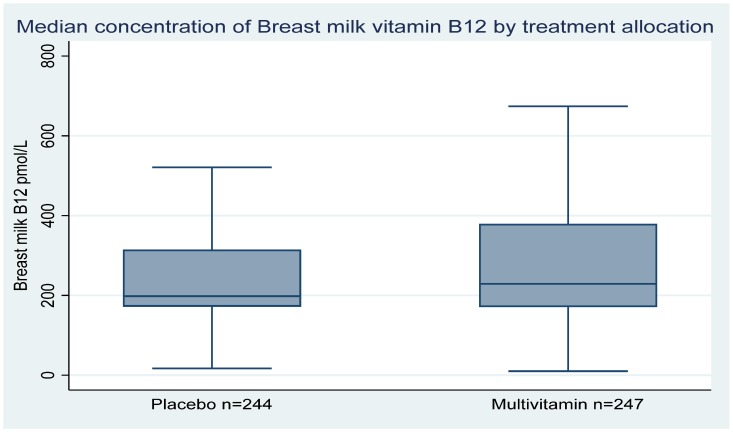
Boxplots comparing the median breast milk vitamin B12 concentrations at 6^th^ week postpartum visit between women in the multivitamin and placebo groups in Urban Tanzania.

The median concentration of milk vitamin B12 was 229 pmol/L (171, 379 pmol/L) in the multivitamin group and 198 pmol/L (172,314.5) in the placebo group (Figure 2). The use of multivitamin supplements increased the vitamin B12 concentration in breast milk at six weeks compared to the placebo arm. However, the difference between the two arms was not statistically significant (change in B12 concentration = 7.5%, 95% CI = −3.4,18.3, *p* = 0.18) (Table 2). Overall, the proportion of women with vitamin B12 concentrations below 310 pmol/L was 70%. The proportion of women with breast milk B12 less than AI value was non-significantly higher in the placebo group (74%) than in the multivitamin supplement group (67%), *p* = 0.09.

### 3.3. Predictors of Vitamin B12 Status

We noted an inverse association between maternal hemoglobin concentrations at six weeks postpartum and the risk of vitamin B12 less than AI value in breast milk. The odds of having milk vitamin B12 less than AI value decreased by 18% (OR= 0.82, 95% CI (0.72, 0.94) for every 1 g/dL increase of hemoglobin at the six weeks postpartum visit (Table 3).

We examined predictors of the continuous concentration of vitamin B12, and noted a similar trend, with an increase of 3.7% for every 1 unit (g/dL) increase in hemoglobin at 6 weeks postpartum (95% CI = 0.5, 6.9, *p* = 0.02).

Participants with baseline BMI between 22.0–24.9 kg/m^2^ and 25.0–29.9 kg/m^2^ had double the odds of having lower vitamin B12 in breast milk compared to those who had baseline BMI below 22 kg/m^2^: OR = 1.92 (95% CI: 1.11,3.33, *p* = 0.02) and 1.91 (1.09,3.36, *p* = 0.03), respectively.

Dietary protein and vitamin B12 intakes were not associated with breast milk B12 concentration, defined either as a continuous variable or a categorical outcome. In multivariate analyses, the tests for trend across tertiles of intake were not statistically significant for dietary vitamin B12 (*p* = 0.86), total protein intake (*p* = 0.67), or animal source protein (*p* = 0.78) (Table 3).

## 4. Discussion

The proportion of women with inadequate breast milk vitamin B12 concentrations in our study of lactating mothers residing in urban Tanzania was 70% despite the reported high dietary intake of vitamin B12 and high adherence to maternal multivitamin supplementation containing vitamin B12. Adherence to the use of multivitamin supplements in the primary study was 80% (median, 86%) for the period from randomization to six weeks postpartum [10]. High prevalence of breast milk vitamin B12 concentrations less than AI value (310 pmol/L) has also been reported from rural Kenya reaching 89% [9], and Cambodia where low concentrations below 362 pmol/L were detected in 75% of lactating women [4]. Since breast milk samples were collected between 1 and 6 months postpartum in Kenya, and between 3 and 27 weeks (mean = 15 weeks) in Cambodia, the progressive postpartum decline in breast milk vitamin B12 concentration [7] could explain the high prevalence of deficiency found in Kenya and Cambodia as compared to the early postpartum samples in Tanzania.

Multivitamin supplements containing 50µg of vitamin B12 produced a non-statistically significant decline in the odds of having vitamin B12 inadequacy in breast milk. These findings are different from trials in India and Bangladesh [6,8], which reported increases in median breast milk vitamin B12 concentration following oral supplementation with varying doses of vitamin B12. The trial in India which used 50µg of vitamin B12 supplements was conducted among malnourished women with a high prevalence of vitamin B12 deficiency because of low intake of vitamin B12 for cultural reasons (vegetarianism), as opposed to the women in our study in urban Tanzania who had better nutritional status in terms of BMI and with a median dietary intake of vitamin B12 above the RDA. The absolute level of intake in Tanzania may not be adequately assessed in our sample given likely measurement errors associated with dietary assessment instruments. Yet we noted poor dietary diversity, as indicated by very low minimum dietary diversity score, suggesting overall lower intake of animal source foods and poor micronutrient status. Insufficient liver stores before pregnancy [9], and malabsorption due to chronic gastritis or Helicobacter pylori [16], may also explain the deficiency status during lactation [16].

In the trial conducted in Bangladesh (n = 68), the dose of oral vitamin B12 supplements used during pregnancy was five times that used in the Tanzania study (250 µg/day), and it resulted in a statistically significant increase of median vitamin B12 concentration in colostrum [8]. At 3 months of lactation, this higher dose of vitamin B12 supplementation produced higher median concentrations of milk B12 (235 pmol/L) compared to the placebo, with a good safety profile [8]. Prospective trials to determine the optimal dose of vitamin B12 supplementation during pregnancy and lactation need to account for the nutritional status of women, the baseline vitamin B12 status in the study population, duration of supplementation, and social-cultural reasons affecting the intake of food containing vitamin B12 [17].

We found that recent intake of dietary vitamin B12 and animal protein did not affect the concentration of B12 in breast milk. The finding is consistent with results obtained in a sub study of women enrolled in a cluster-randomized trial to evaluate the efficacy of Water Sanitation and Hygiene (WASH) interventions on child growth in Kenya [9]. These findings may be explained by the fact that maternal physiology may give preference to the restoration of vitamin B12 stores in the liver rather than recruitment of B12 in mammary glands in B12 deficient lactating mothers [9]. It is also possible that the measurement error of dietary intake using the FFQ that we have administered may have biased a true association between dietary intake and breast milk vitamin B12 concentrations toward the null.

In our study, higher BMI at baseline was associated with lower concentration of vitamin B12 in breast milk which is in line with studies in India [18] and the United Kingdom [19]. In the UK study, the association of lower serum B12 with higher maternal BMI and insulin resistance was highly significant even after controlling for confounding effects of demographic and lifestyle factors. It has been hypothesized that maternal obesity and associated changes in body fat distribution and metabolism could result in the lowering of circulating levels of vitamin B12 [19]. Similar results were reported from India, where maternal vitamin B12 deficiency was associated with increased insulin resistance and gestational diabetes mellitus [18].

We also noted that hemoglobin concentration at six weeks postpartum was positively associated with breast milk vitamin B12. This finding may indicate the importance of maternal B12 levels in the maintenance of normal hematologic status. Vitamin B12 is essential for proliferation and maturation of erythroblasts, and its deficiency, impairs the synthesis of DNA, causing the death of erythroblasts resulting in anemia [20]. Previous studies that evaluated the concentration of B12 and folate during pregnancy in relation to gestational stage and hemoglobin levels have shown that there are increased demands for vitamin B12 in the first 27 weeks and in the third trimester of pregnancy [21].

Our study had several limitations. The cross-sectional measurement of exposure variables during pregnancy prevents examination of causality between predictors and vitamin B12 levels in breast milk. We also relied on a single measurement at six weeks postpartum to estimate the effects of multivitamin supplements on the concentration of B12 in breast milk. The repeated assessment of breast milk vitamin B12 concentration at multiple time points during the follow-up period could have improved the evaluation of effects of multiple micronutrient supplements on B12 in milk. The generalizability of the study findings is affected by the fact that the primary study was only conducted among HIV-negative urban women of African descent and hence not applicable to HIV infected or rural women or other ethnic backgrounds in Tanzania. Additionally, there was no information about gestational diabetes mellitus (GDM) or preeclampsia in the primary study which could indirectly affect the maternal vitamin B12 status and secretion of vitamin B12 in breast milk. We could only analyze 500 breast milk samples and therefore the study was insufficiently powered to detect changes in breast milk vitamin B12 concentration between the multivitamin supplements and placebo groups.

However, notable strengths include the long duration of supplementation and high adherence to supplementation allowing for meaningful interpretation of the effect of multivitamin supplements on milk vitamin B12 concentration at six weeks postpartum. Another strength lies in the use of an accurate method for the measurement of breast milk vitamin B12 concentration using the IMMULITE method [14,17].

The use of multiple regression methods to control for a number of possible confounding variables that further enhance the secondary observational analysis such as socio-demographic, dietary intake, and pregnancy characteristics.

## 5. Conclusions

Multiple micronutrient supplements containing 50 µg of vitamin B12 resulted in a modest increase in breast milk concentrations of vitamin B12 that was not statistically significant. In light of high proportion of mothers that remained with breast milk B12 concentrations at less than AI in this population, randomized controlled trials are needed to determine the optimal dose, route of administration, and the duration of supplementation before recommending vitamin B12 supplementation as an additional strategy to improve maternal and infant outcomes in sub-Saharan Africa.

## Figures and Tables

**Table 1:** Baseline characteristics of postpartum mothers included in the study of 6-weeks postpartum breast milk concentration among HIV-negative women in Dar es Salaam, Tanzania.

Variable	Multivitamin (*N* = 247)	Placebo (*N* = 244)
Socio-demographic characteristics		
Maternal education (years) *n* (%)		
0–4	27(10.9)	28(11.5)
5–7	156(63.2)	154(63.1)
8–11	40(16.2)	46(18.9)
12+	22(8.9)	14(5.7)
Marital status *n* (%)		
Unmarried	21(8.5)	30(12.3)
Married/Co-habiting	222(89.9)	211(86.5)
Wealth score *n* (%)		
At or above median	138(55.9)	133(54.5)
Below median	106(42.9)	109(44.7)
Minimum Dietary Diversity Score (MDD-W) *n* (%)		
Less than 5 groups	238(99.6)	233(97.9)
5 or more groups	1(0.4)	5(2.1)
Dietary intake median (IQR)		
Calorie intake (kcal/day)	2264.9(1790,2675)	2273.6(1806,2753)
Dietary Vit B12 intake (µg/d) median (IQR)	2.9(1.5,8.4)	3.4(1.4,8.0)
Tertiles of B12 intake (µg/d) *n* (%)		
1^st^ (Less than 1.85)	83(33.6)	74(30.3)
2^nd^ (1.85–5.97)	74(30.0)	83(34.0)
3^rd^ (more than 5.97)	84(34.0)	77(31.6)
Total protein intake (g/d) median (IQR)	82.5(59.8,115.3)	81.9(59.2,104.6)
Tertiles of total protein intake(g/d) *n* (%)		
1^st^ (Less than 65.5)	77(31.2)	80(32.8)
2^nd^ (65.5–98.9)	80(32.4)	77(31.6)
3^rd^ (more than 98.9)	84(34.0)	77(31.6)
Animal protein intake (g/d) median (IQR)	29.5(20.1,43.5)	30.2(19.5,44.1)
Tertiles of animal protein (g/d) *n* (%)		
1^st^ (Less than 22.8)	77(31.2)	81(33.2)
2^nd^ (22.8–37.0)	81(32.8)	76(31.2)
3^rd^ (more than 37.0)	83(33.6)	77(31.6)
Pregnancy Characteristics		
Gestational age at enrolment (weeks) mean (SD)	21.4(3.2)	21.4(3.2)
Gestational age at enrolment *n* (%)		
At or beyond 20 weeks	167(67.6)	163(66.8)
Less than 20 weeks	80(32.4)	81(33.2)
Biochemical data		
Breast milk B12 (pmol/L) median (IQR)	229(171,379)	198(172,314.5)
Baseline hemoglobin (g/dL) mean (SD)	10.4(1.3)	10.3(1.4)
Hemoglobin at 6 weeks (g/dL) mean (SD)	12.3(1.9)	12.0(1.5)
Hb categories at 6 weeks (g/dL) *n* (%)		
<8.5	10(4.1)	5(2.1)
8.5–10.9	31(12.6)	47(19.3)
>=11.0	202(81.8)	188(77.1)
Baseline anthropometry		
Body mass index(BMI) mean (SD)	25.1(3.9)	24.4(3.9)
Baseline categories of BMI (kg/m^2^) *n* (%)		
<22.0	50(20.2)	70(28.7)
22.0–24.9	72(29.2)	75(30.7)
25.0–29.9	80(32.4)	58(23.8)
>=30	24(9.7)	21(8.6)
Pregnancy History		
Parity *n* (%)		
0	93(38.0)	101(41.7)
1	70(28.6)	76(31.4)
2	41(16.7)	36(14.9)
3+	41(16.7)	29(12.0)

Percent may not add to 100% because of rounding and missing numbers.

**Table 2:** Effect of multivitamin supplements containing 50 mcg of Vitamin B12 concentration on breast milk vitamin B12 concentration among HIV-negative women at 6-weeks postpartum in Dar es Salaam Tanzania.

Study Regimen	Breastmilk B12 Concentration (Linear B12) in pmol/L ^1^				Breastmilk B12 Categories (B12 < 310 pmol/L = AI) ^2^			
	Univariate Analysis (*N* = 491)		Multivariate Analysis (*N* = 491) ^3^		Univariate Analysis (*N* = 491)		Multivariate Analysis (*N* = 491) ^4^	
	Percent Change in B12 Concentration and 95% CI	*p*-Value	Percent Change in B12 Concentration and 95% CI	*p*-Value	Odds Ratio and 95% Confidence Intervals	*p*-Value	Odds Ratio and 95% Confidence Intervals	*p*-Value
Placebo	Ref		Ref		Ref		Ref	
Multivitamin supplements	7.1(−3.7,18.0)	0.20	7.5(−3.4,18.3)	0.18	0.72(0.48,1.06)	0.09	0.73(0.49,1.09)	0.12

^1^ Based on linear regression with log (B12 concentration) as the dependent variable. Coefficients and 95% confidence limits have been exponentiated to compute the percent changes in B12 concentration. ^2^ Based on logistic regression with vitamin B12 deficiency defined by vitamin B12 concentration less than 310 pmol/L and concentrations at or above 310 pmol/L (adequate intake) as a categorical outcome. The odds ratio (OR) are expressed together with their 95% confidence intervals in brackets. ^3^ Multivariable model adjusted for the intake of animal protein, maternal education, marital status, gestational age at enrolment before 20 weeks, maternal hemoglobin concentration, parity, and baseline body mass index (BMI). ^4^ Multivariable model adjusted for the intake of total calories, vitamin B12, total and animal protein, gestational age at enrolment before 20 weeks, maternal hemoglobin concentration, and baseline BMI at recruitment.

**Table 3:** Predictors of milk B12 less than 310 pmol/L at 6-weeks postpartum among HIV-negative women in Dar es Salaam, Tanzania.

		Univariate ^1^		Multivariate (N = 491) ^3^	
Variable	N^2^	Odds Ratio and 95% CI	*p*-Value	Odds Ratio and 95% CI	*p*-Value
Socio-demographic characteristics					
Maternal education (Years)					
0–4		Ref			
5–7		0.99(0.53,1.86)			
8–11	491	1.19(0.56,2.54)			
12+		0.57(0.24,1.39)			
		P for trend	0.36		
Marital Status					
Unmarried		Ref			
Married/Co-habiting	491	0.98(0.52,1.86)	0.96		
Wealth score					
At or above median		Ref			
Below median	491	1.16(0.78,1.72)	0.46		
					
Dietary intake					
Calorie intake (kcal/day)	491	1.00(0.99,1.00)	0.08	1.00(0.99,1.00)	0.12
Dietary Vit B12 intake (µg/d)	491	1.00(0.98,1.02)	0.64	1.00(0.98,1.03)	0.86
Tertiles of Vit B12 intake (µg/d)					
1^st^ (Less than 1.85)		Ref			
2^nd^ (1.85–5.97)	491	1.17(0.72,1.91)			
3^rd^ (more than 5.97)		0.92(0.57,1.48)			
		P for trend	0.48		
Dietary protein intake (g/d)	491	1.00(0.99,1.01)	0.45	1.00(0.99,1.01)	0.67
Tertiles of dietary protein intake (g/d)					
1^st^ (Less than 65.5)		Ref			
2^nd^ (65.5–98.9)	491	1.27(0.79,2.06)			
3^rd^ (more than 98.9)		1.32(0.81,2.13)			
		P for trend	0.53		
Animal protein intake (g/d)	491	1.00(0.99,1.00)	0.44	1.00(0.98,1.020)	0.78
Tertiles of animal protein intake (g/d)					
1^st^ (Less than 22.8)		Ref			
2^nd^ (22.8–37.0)	491	0.79(0.49,1.26)			
3^rd^ (more than 37.0)		1.36(0.83,2.24)			
		P for trend	0.53		
Pregnancy Characteristics					
Gestational age at enrolment (weeks)	491	1.03(0.97,1.09)	0.32		
Gestation age at enrolment					
At or beyond 20 weeks		Ref		Ref	
Less than 20 weeks	491	0.67(0.45,1.01)	0.06	0.74(0.49,1.13)	0.16
Biochemical data					
Hemoglobin at 6 weeks in g/dL	491	0.80(0.70,0.91)	<0.01	0.82(0.72,0.94)	<0.01
Hemoglobin categories at 6 week (g/dL)					
<8.5		Ref			
8.5–10.9	491	0.45(0.09,2.15)			
>=11.0		0.34(0.08,1.54)			
		P for trend	0.59		
Baseline anthropometry					
BMI (continuous) in kg/m^2^	491	1.02(0.97,1.07)	0.45		
Baseline BMI (Categorical) in (kg/m^2^)					
<22.0		Ref		Ref	
22.0–24.9		1.60(0.95,2.71)	0.08	1.92(1.11,3.33)	0.02
25.0–29.9	491	1.52(0.90,2.59)	0.12	1.91(1.09,3.36)	0.03
>=30		1.12(0.54,2.30)	0.77	1.56(0.73,3.35)	0.26
		P for trend	0.80	P for trend	0.73
Pregnancy History					
Parity					
0		Ref			
1		0.74(0.47,1.18)			
2	487	0.91(0.51,1.62)			
3+		1.20(0.64,2.26)			
		P for trend	0.72		

^1^ Based on logistic regression with less than adequate intake (AI) defined by milk vitamin B12 concentrations less than 310 pmol/L and concentrations at or above 310 pmol/L (adequate intake) as a categorical outcome. The odds ratios (OR) are expressed together with their 95% confidence intervals in brackets. ^2^ This column shows the number of women for whom the variable was non-missing. When variables with missing values were included in the multivariate model, missing indicators were used. Data on parity was unavailable for 4 women (0.8%). ^3^ The multivariate model included calorie intake, intake of vitamin B12, total and animal protein, gestational age at enrolment below 20 weeks, hemoglobin at 6 weeks postpartum as continuous variables, and BMI at recruitment.
